# Supplementary material for: Patterns of Intron Gain and Loss in Fungi
Source: PLoS Biol. 2004 Nov 30;2(12):e422. doi: 10.1371/journal.pbio.0020422 (PMC532390; doi:10.1371/journal.pbio.0020422)
Supplement: Table S1 — Also available at http://genes.mit.edu/NielsenEtAl/. (4.3 MB ZIP). [file pbio.0020422.st001.zip › NielsenEtAl/html/734.html]

AN6922.1.NCU01795.1.MG07010.1.FG10829.1


```
 CLUSTAL W (1.82) Multiple Sequence Alignments - Introns Inserted


Sequence 1: NCU01795.1	355 aa
Sequence 2: MG07010.1	345 aa
Sequence 3: FG10829.1	358 aa
Sequence 4: AN6922.1	340 aa
Alignment Length: 362 aa
Number Identitical Residues: 208 aa
Alignment Score (without introns) 9492


MG07010.1 	MPPKKND-KAGGKKPSVTKVVDDK~TFGMKN~KKGGAAQKQIAQLQAQAKSGGNAEQKRK
NCU01795.1	MPPKKQEPKAAGKK----KAVEDK~TFGMKN~KKGSAAQKQIAALQASMKNGGNAEQKRK
FG10829.1 	MAPPKKGKEPQPKK--ATKTVEDR~TFGMKN~KKGGAAQREIGRMQANLKNGGTAEEKKK
AN6922.1  	MPPKKPD-QPKKKK----ATVDDK0TFGMKN0KKGGSAKKQIAQLQAQAAHSKTADEKRK
          	*.* *   :.  **     .*:*: ****** ***.:*:::*. :**.   . .*::*:*

MG07010.1 	EAEKAAREREKKAAEDAKREADLLLNKPAQIQKVPFGVDPKTVLCIFFKKGNCEKGKKCK
NCU01795.1	EAEKAAREREKKAAEDAKREADLLLNKPAQIQKVPFGVDPKTVVCIFFKKGNCEKGKKCK
FG10829.1 	QAEKAAREREKKAAEDAKRDMEAMINRPAQIQKVPFGVDPKTVVCIFYKKGNCEKGKKCK
AN6922.1  	AAEKERREAEKRAAEQAKKEALELF-KPVQVQKVPFGVDPKTVLCQFYKQGHCEKGRKCK
          	 ***  ** **:***:**::   :: :*.*:************:* *:*:*:****:***

MG07010.1 	FSHDLDVERKTEKKSLYTDTRENEDDKKKAETSADWDEDKLRSVVLSKKGNQRTTTDKVC
NCU01795.1	FSHDLEQERKVEKRNLYQDTRAEEDDKKKQETSADWDEEKLRSVVLSKKGNQRTTTDKVC
FG10829.1 	FSHDLSVERKTEKKNLYTDKRGEEEETKKVETSADWDEDKLRSVVLSKKGNQQTTTDKVC
AN6922.1  	FSHDLNVDRKAAKKDLYTDSREAEDEKKKADTMEQWDEAKLRSVVLSKHGNPRTTTDKVC
          	*****. :**. *:.** *.*  *::.** :*  :*** *********:** :*******

MG07010.1 	KYFIEAIEDGKYGWFWICPNGGDKCMYKHALPQG2FVLKTKEDRAREKALLDKSPLKTLT
NCU01795.1	KFFIEAIEEGKYGWFWICPNGGDKCMYKHALPPG2FVLKTKEQRAAEKALLDKSPLRTLT
FG10829.1 	KFFIEAIEDGKYGWFWICPNGGDKCKYKHALPPG2FVLKTKEQRAAEKALMDKSPLKTLT
AN6922.1  	KYFIEAVENQKYGWFWVCPNGGDKCMYKHSLPPG2FVLKTKEQRAAEKAMMDKSPLNTLT
          	*:****:*: ******:******** ***:** * *******:** ***::*****.***

MG07010.1 	LEEFLESERHKLTGTLTPVTPESFAKWKKDRLDKKAAEEQARKAKDATGRALFESGNWRT
NCU01795.1	LEEFLESERHKLTGTLTPVTPESFAKWKKERLDKKAAEEAARKAKEATGRALFESGNWRM
FG10829.1 	LEDFLESERHKLTGTLTPVTPESFAKWKKDRLDKKAAEEQARNAKENTGRAMFESGKWRD
AN6922.1  	LEDWLESERHKLTGELTPVTPETFAKWKKERMDKKAAEEQARKAKEATGRTLFESGNWRT
          	**::********** *******:******:*:******* **:**: ***::****:** 

MG07010.1 	EEDDD-DAEDDDDPWNLEKLRAETEAIRQRKEEERLAIENGVT---VPG-----------
NCU01795.1	EADIDSDDEGDDDAWNLEKLRKETEAIQIKKEEERLSALHGVP---VTLGEAKDVTGLSE
FG10829.1 	EDDSD--SEDDDDVWNLQKLREETENIRSKKEEERLMGSYETPNNGTTGQATPAEDTPAE
AN6922.1  	EESEEESDEDDDGAWNLAALRKETERIREQKEEERLAKLHGEQ---QPGGED--------
          	* . :.. *.**. ***  ** *** *: :******           .            

MG07010.1 	-APGT
NCU01795.1	PTLGT
FG10829.1 	EASGS
AN6922.1  	-----
          	
```
